# Zonisamide: A Comprehensive, Updated Review for the Clinician

**DOI:** 10.1212/CPJ.0000000000200210

**Published:** 2023-11-10

**Authors:** Barry E. Gidal, Trevor Resnick, Michael C. Smith, James W. Wheless

**Affiliations:** Pharmacy Practice & Translational Research (BEG), University of Wisconsin-Madison; Department of Neurology (TR), Nicklaus Children Hospital; Department of Neurology, Florida International University, Miami, FL; Department of Neurological Sciences (MCS), Rush Medical College; Rush University Medical Center, Chicago, IL; and Pediatric Neurology (JWW), University of Tennessee Health Science Center; Neuroscience Institute & Le Bonheur Comprehensive Epilepsy Program, Le Bonheur Children's Hospital, Memphis, TN.

## Abstract

**Purpose of Review:**

Zonisamide (ZNS) was first approved in the United States in 2000 for the adjunctive treatment of patients aged 16 years or older with partial (focal) seizures. Although ZNS has been proven to treat multiple seizure types, it has been largely underutilized in US clinical practice.

**Recent Findings:**

Published literature demonstrated that antiseizure medications (ASMs) acting on Na^+^ and Ca^2+^ channels may add beneficial effects in many seizure types by reducing seizure frequency and leading to overall improvements. In addition, effects of ZNS may lead to clinical improvements in Parkinson disease, alcohol and sleep disorders, pain, and migraine. ZNS is available in multiple formulations and is a safe and effective, broad spectrum ASM.

**Summary:**

The purpose of this review was to provide an update to what is known about the efficacy of ZNS and where it shows benefits in the treatment of patients with epilepsy and other CNS disorders through its many unique mechanisms of action.

## Introduction

### History

**Figure 1:**
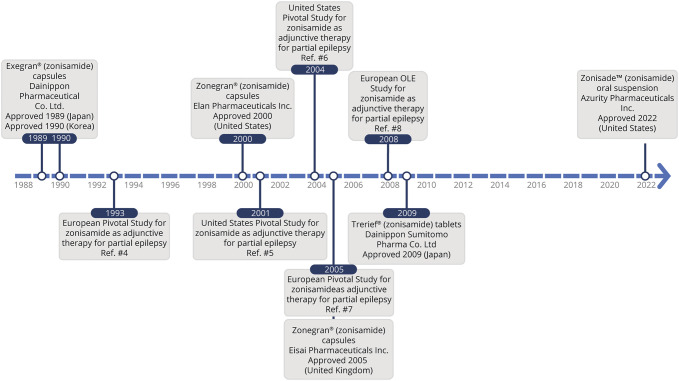
Timeline of ZNS Approval and Its Pivotal Trials Across the Globe

Zonisamide (ZNS) was first discovered in 1974 from routine testing of benzisoxazole analogs originally researched for psychiatric disorder purposes.^1^ ZNS was synthesized in 1979 for its antiseizure properties, approved in Japan in 1989, and then approved in the United States in 2000, a few months after levetiracetam was launched.^1,2^ ZNS was sold in 2002 to Eisai Co., Ltd., before evidence of its full benefits in epilepsy, and other CNS disorders were appreciated.^3^ After its discovery, ZNS was tested in multiple animal models and was shown to exhibit anticonvulsant activity as potent as phenobarbital and carbamazepine (CBZ) but greater than phenytoin (PHT).^1^ Since then, ZNS has been approved with multiple formulations spanning many countries (Figure 1).

Since 1989, ZNS has been studied in multiple Japanese clinical trials and has demonstrated efficacy against simple and complex partial seizures (now referred to as focal seizures without or with impaired awareness), partial seizures with secondary generalization (referred to as focal seizures progressing to bilateral tonic-clonic seizures), and generalized seizures (tonic-clonic, tonic, clonic, atonic, myoclonic, typical absence, and atypical absence seizures).^3^ Since then, several global studies have been conducted to evaluate the efficacy of ZNS. CBZ, an older antiseizure medication (ASM), was used historically as an efficacy standard for focal seizures, and newer ASMs were typically compared with this in randomized controlled trials. Through comparative studies against gabapentin (GBP), lamotrigine (LTG), levetiracetam (LEV), valproate (VPA), topiramate (TPM), ZNS, oxcarbazepine (OXC), eslicarbazepine (ESL), and lacosamide (LCS), CBZ was found to be significantly more effective or as effective than comparator ASMs for multiple seizure types.^4^ ZNS was determined to be as effective as CBZ with favorable long-term safety and maintenance of efficacy for treating focal seizures in adults.^5,6^ Although there are few clinical trials examining ZNS against other ASMs, its relative efficacy compared with CBZ suggests meaningful efficacy when compared with the lesser efficacy of other ASMs (GBP, TPM).^7^ Although ZNS has been proven to treat multiple seizure types with efficacy comparable with other ASMs, it has been largely underutilized in US clinical practice. This review aims to provide a comprehensive overview of current information/findings on ZNS and additional opportunities to broaden its use and benefits in the treatment of epilepsy and other disease states.

### Mechanism of Action

**Figure 2:**
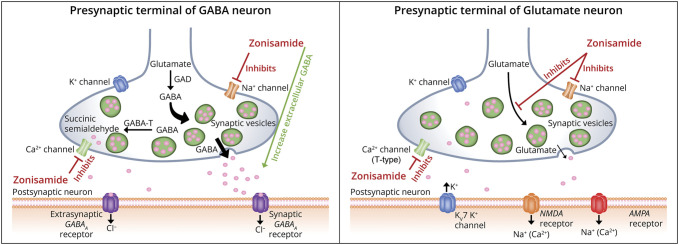
MOA for ZNS to Target Multiple Pathways

ZNS is a benzisoxazole analog (1,2-benzisoxazole-3-methanesulfonamide) with proposed multiple mechanisms of action (MOAs), including inhibitory effects on voltage-gated sodium and T-type calcium channels, predicting effectiveness in generalized tonic-clonic and absence seizures.^8^ Blockade of sustained, repetitive firing through voltage-sensitive sodium, and T-type calcium channels are likely the principle antiseizure mechanisms. In addition, ZNS may inhibit presynaptic glutamate release and possibly enhance γ-aminobutyric acid (GABA) function by influencing GABA transport (Figure 2). In mice models, ZNS was shown to decrease nitric oxide levels in a dose-dependent fashion.^9^ Nitric oxide modulates many brain functions and is involved in the pathogenesis of convulsive seizures by promoting neuronal synchronization. Although ZNS has a sulfamoyl side chain group (similar to acetazolamide), it does not seem to exert significant carbonic anhydrase activity, so this is an unlikely contributor to its antiseizure actions. ZNS's unique ability to target multiple pathways makes it an ideal alternative/adjunctive option for patients with epilepsy refractory to other ASMs.^10^ Adding ZNS as adjunctive treatment with other ASMs may permit complementary MOAs to improve efficacy without tolerability issues.

### Pharmacokinetics—Practical Implications

ZNS is readily and rapidly absorbed, with peak concentrations occurring within 2–4 hours and has a high bioavailability (Table 1).^11-13^ Zonisamide is not significantly bound to plasma proteins (∼40%), but interestingly, it does seem to concentrate in erythrocytes (perhaps up to 8-fold higher RBC concentrations vs plasma) in a linear fashion. The ultimate clinical impact of this is unclear. Multiple clinical trials show that ZNS serum trough concentrations (between 10 and 40 mg/L) correspond to clinical efficacy. A long elimination half-life (∼50–60 hours) because of relatively lower systemic clearance suggests that ZNS may be an ideal therapeutic option for once-daily dosing, which may improve medication adherence. Patient adherence can be heavily influenced by a convenient medication dose and a simple medication regimen.^2^ Lack of patient adherence to medication regimens often leads to seizures. Contrary to other second-generation or third-generation ASMs prescribed for focal seizures with a twice-daily maintenance schedule (Table 1),^11-13^ ZNS may allow patients greater ease of use and clinical outcomes due to once-daily dosing and potential for increased medication adherence.^2,14-16^

**Table 1:**
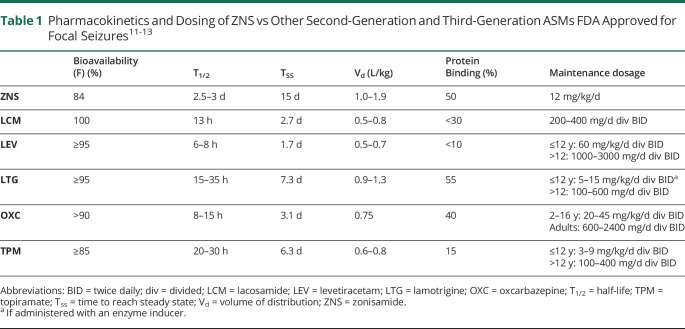
Pharmacokinetics and Dosing of ZNS vs Other Second-Generation and Third-Generation ASMs FDA Approved for Focal Seizures^11-13^

	Bioavailability (F) (%)	T_1/2_	T_SS_	V_d_ (L/kg)	Protein Binding (%)	Maintenance dosage
ZNS	84	2.5–3 d	15 d	1.0–1.9	50	12 mg/kg/d
LCM	100	13 h	2.7 d	0.5–0.8	<30	200–400 mg/d div BID
LEV	≥95	6–8 h	1.7 d	0.5–0.7	<10	≤12 y: 60 mg/kg/d div BID >12: 1000–3000 mg/d div BID
LTG	≥95	15–35 h	7.3 d	0.9–1.3	55	≤12 y: 5–15 mg/kg/d div BID^a^ >12: 100–600 mg/d div BID
OXC	>90	8–15 h	3.1 d	0.75	40	2–16 y: 20–45 mg/kg/d div BID Adults: 600–2400 mg/d div BID
TPM	≥85	20–30 h	6.3 d	0.6–0.8	15	≤12 y: 3–9 mg/kg/d div BID >12 y: 100–400 mg/d div BID

Abbreviations: BID = twice daily; div = divided; LCM = lacosamide; LEV = levetiracetam; LTG = lamotrigine; OXC = oxcarbazepine; T_1/2_ = half-life; TPM = topiramate; T_ss_ = time to reach steady state; V_d_ = volume of distribution; ZNS = zonisamide.

a If administered with an enzyme inducer.

ZNS is hepatically metabolized both by acetylation as well as reduction to a 2-sulfamoylacetyl phenol metabolite that is mediated by CYP3A4. This CYP450 metabolism is subject to drug interactions between enzyme-specific inducers and inhibitors.^14^ However, ZNS does not inhibit cytochrome P450 isozyme nor UDP-glucuronyl transferase (e.g., glucuronidation), so it does not affect serum levels of other ASMs.^1^ Other medications may be coadministered with ZNS without dose adjustments. Alternatively, PB, PHT, primidone (PRM), and CBZ all modulate hepatic metabolic pathways through the CYP isozyme, resulting in dramatically lower ZNS serum levels, when coadministered, requiring higher ZNS dosage.^10^ ASMs may also induce metabolism of estrogen and/or progesterone, possibly leading to hormonal contraceptive failures.^13^ ZNS does not interact with oral contraceptives containing ethinylestradiol and norethindrone, whereas PB, PHT, CBZ, and OXC may reduce contraceptive efficacy.^10,14^ Prescribers must carefully consider ASM-related interactions if patients are also prescribed oral contraceptives, steroids, antibiotics, antifungals, anticoagulant medications, statins, certain antineoplastics, or antiretrovirals. ZNS's lack of pharmacokinetics (PK)–related interactions, coupled with long half-life (t_1/2_) for once-daily dosing, may provide an advantage over ASMs with considerable drug interactions and increased dosing frequency.

### ZNS Concentration-Dependent Effects

ZNS maintains a dose and drug concentration relationship in adults and pediatrics based on animal and human studies.^1,3,10,14^ With single doses of ZNS 100–800 mg/d, there is a linear increase in maximum serum concentration (C_max_) and area under the plasma concentration curve (AUC). This linear trend of dose and an efficacy of responder rate (*p* < 0.0001) were demonstrated in a study of ZNS 300 or 500 mg/d vs placebo.^14^ However, concomitant administration with other ASMs could lead to reduction of ZNS serum concentrations—such patients may benefit with higher doses of >600 mg/d. Providers may underestimate ZNS efficacy when used as adjunctive therapy to other ASMs; dosages may not be appropriate, leading to inadequate ZNS serum concentration levels. Clinicians should be mindful of the potential impact of concomitant treatment with CYP inducing medications and dose to effect. The assessment of ZNS serum levels can help guide dose adjustments.

Despite its advantages (ideal PK profile, convenient dosing schedule, and history of efficacy/safety as mono/adjunctive therapy for various seizures for over 34 years), ZNS may not have been widely prescribed because of its perceived barriers to cause adverse effects (AEs) such as renal calculi and metabolic acidosis. There is a missed opportunity to use ZNS as a broad spectrum agent in many different patient populations.

### US Guidelines: Role of ZNS in Epilepsy

The 2004 American Academy of Neurology (AAN) and the American Epilepsy Society (AES) guidelines determined ZNS to be an established and effective treatment recommendation for adjunctive therapy in adults with treatment-resistant (TR) focal epilepsy (Level A) but determined that additional studies were needed to substantiate the role of ZNS for pediatric patients with TR epilepsy (Level U).^15^ However, in 2018, new data allowed AAN and AES to expand the use of ZNS for the treatment of pediatric patients (ages 6–17 years) with TR focal epilepsy (Level B).^16^ Other second-generation and third-generation ASMs (clobazam [CLB], eslicarbazepine [ESL], lacosamide [LCS], perampanel [PER], pregabalin [PGB], rufinamide [RFM], tiagabine [TGB], and vigabatrin [VGB]) did not have efficacy data for use in pediatric patients with TR focal epilepsy compared with ZNS (Level U). At this time, cenobamate was not yet available. The 2018 AAN/AES expert subcommittee also did not document any new serious safety concerns associated with ZNS since the 2004 guidelines.

The updated 2018 AAN/AES guidelines provided evidence to support the use of ZNS to reduce seizure frequency in both new-onset and TR focal epilepsy.^4,16^ A phase 3, randomized, double-blind, noninferiority trial suggested that ZNS could be useful as initial monotherapy for patients with newly diagnosed focal epilepsy and may be considered to decrease seizure frequency (Level C), in contrast to third-generation ASMs (e.g., CLB, felbamate, or VGB), which do not have data for use in these populations.^5,16^ More data and research were deemed necessary to determine cost-effectiveness of ZNS and its efficacy in treating newly diagnosed epilepsy in children.

## Literature Search Strategy (Efficacy of ZNS)

Data from 2004 to 2022 were collected from PubMed using specified search criteria focusing on the efficacy and safety of ZNS. Search terms included “zonisamide,” “epilepsy,” “partial seizures,” “focal seizures,” “general epilepsy,” “childhood epilepsy,” and “efficacy.” Publications involving animal studies, PK studies, phase 1–2 studies, studies not published in English, or studies with sample size (N) < 15 were excluded. Safety data were evaluated within the literature findings of ZNS efficacy studies. The remaining articles were reviewed for relevance using select criteria from the 2010 CONSORT and 2020 PRISMA guidelines (eAppendix 1, links.lww.com/CPJ/A475), including assessment of the study methods (i.e., sample size, eligibility criteria, randomization), data collection process, discussion, and availability of data.

## Efficacy of ZNS in Epilepsy

ZNS stabilizes neuronal membranes through Na^+^ and Ca^2+^ channels to reduce epileptiform activity.^8^ ASMs modulating Na^+^ and Ca^2+^ channels are relevant in focal, generalized tonic-clonic (GTC), and absence seizures. Overall, ZNS has a linear relationship between a dose of ≤13 mg/kg/d and serum ZNS concentrations in adult and pediatric participants. As discussed above, data suggest a concentration-effect relationship for this medication which may have an advantage in optimizing therapeutic effect.^1,14^

In both pediatric and adult patients, ZNS has been studied in focal, GTC, absence, and childhood epilepsies as a new-onset or refractory treatment option. Table 2^5,6,17^ summarized ZNS studies in adult patients only with focal and generalized seizures (no other studies fitting our search parameters evaluated adults only in different seizure etiologies), whereas Table 3^18-30^ summarized studies using ZNS in various seizure etiologies in adult and pediatric patients. Randomized controlled trials (RCTs), open-label extension studies (OLEs), retrospective studies, and postmarketing prospective studies were included in this review.

**Table 2:**
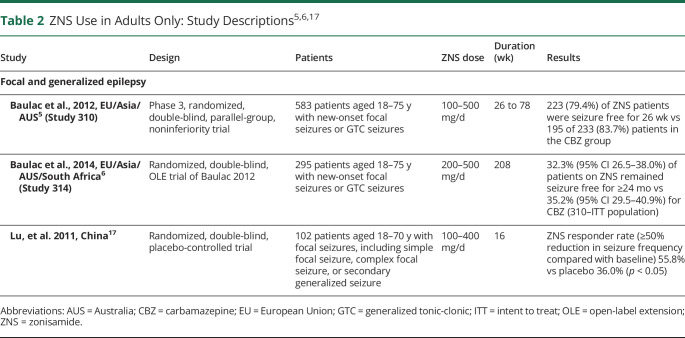
ZNS Use in Adults Only: Study Descriptions^5,6,17^

Study	Design	Patients	ZNS dose	Duration (wk)	Results
Focal and generalized epilepsy					
Baulac et al., 2012, EU/Asia/AUS^5^ (Study 310)	Phase 3, randomized, double-blind, parallel-group, noninferiority trial	583 patients aged 18–75 y with new-onset focal seizures or GTC seizures	100–500 mg/d	26 to 78	223 (79.4%) of ZNS patients were seizure free for 26 wk vs 195 of 233 (83.7%) patients in the CBZ group
Baulac et al., 2014, EU/Asia/AUS/South Africa^6^ (Study 314)	Randomized, double-blind, OLE trial of Baulac 2012	295 patients aged 18–75 y with new-onset focal seizures or GTC seizures	200–500 mg/d	208	32.3% (95% CI 26.5–38.0%) of patients on ZNS remained seizure free for ≥24 mo vs 35.2% (95% CI 29.5–40.9%) for CBZ (310–ITT population)
Lu, et al. 2011, China^17^	Randomized, double-blind, placebo-controlled trial	102 patients aged 18–70 y with focal seizures, including simple focal seizure, complex focal seizure, or secondary generalized seizure	100–400 mg/d	16	ZNS responder rate (≥50% reduction in seizure frequency compared with baseline) 55.8% vs placebo 36.0% (*p* < 0.05)

Abbreviations: AUS = Australia; CBZ = carbamazepine; EU = European Union; GTC = generalized tonic-clonic; ITT = intent to treat; OLE = open-label extension; ZNS = zonisamide.

**Table 3:**
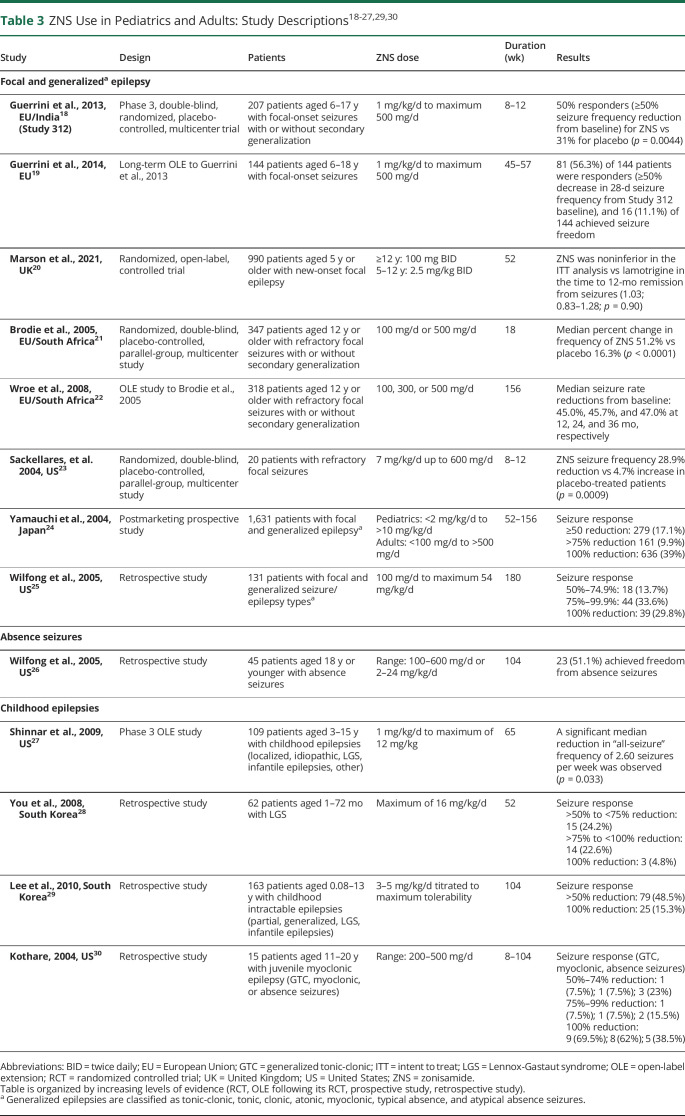
ZNS Use in Pediatrics and Adults: Study Descriptions^18-27,29,30^

Study	Design	Patients	ZNS dose	Duration (wk)	Results
Focal and generalized^a^ epilepsy					
Guerrini et al., 2013, EU/India^18^ (Study 312)	Phase 3, double-blind, randomized, placebo-controlled, multicenter trial	207 patients aged 6–17 y with focal-onset seizures with or without secondary generalization	1 mg/kg/d to maximum 500 mg/d	8–12	50% responders (≥50% seizure frequency reduction from baseline) for ZNS vs 31% for placebo (*p* = 0.0044)
Guerrini et al., 2014, EU^19^	Long-term OLE to Guerrini et al., 2013	144 patients aged 6–18 y with focal-onset seizures	1 mg/kg/d to maximum 500 mg/d	45–57	81 (56.3%) of 144 patients were responders (≥50% decrease in 28-d seizure frequency from Study 312 baseline), and 16 (11.1%) of 144 achieved seizure freedom
Marson et al., 2021, UK^20^	Randomized, open-label, controlled trial	990 patients aged 5 y or older with new-onset focal epilepsy	≥12 y: 100 mg BID 5–12 y: 2.5 mg/kg BID	52	ZNS was noninferior in the ITT analysis vs lamotrigine in the time to 12-mo remission from seizures (1.03; 0.83–1.28; *p* = 0.90)
Brodie et al., 2005, EU/South Africa^21^	Randomized, double-blind, placebo-controlled, parallel-group, multicenter study	347 patients aged 12 y or older with refractory focal seizures with or without secondary generalization	100 mg/d or 500 mg/d	18	Median percent change in frequency of ZNS 51.2% vs placebo 16.3% (*p* < 0.0001)
Wroe et al., 2008, EU/South Africa^22^	OLE study to Brodie et al., 2005	318 patients aged 12 y or older with refractory focal seizures with or without secondary generalization	100, 300, or 500 mg/d	156	Median seizure rate reductions from baseline: 45.0%, 45.7%, and 47.0% at 12, 24, and 36 mo, respectively
Sackellares, et al. 2004, US^23^	Randomized, double-blind, placebo-controlled, parallel-group, multicenter study	20 patients with refractory focal seizures	7 mg/kg/d up to 600 mg/d	8–12	ZNS seizure frequency 28.9% reduction vs 4.7% increase in placebo-treated patients (*p* = 0.0009)
Yamauchi et al., 2004, Japan^24^	Postmarketing prospective study	1,631 patients with focal and generalized epilepsy^a^	Pediatrics: <2 mg/kg/d to >10 mg/kg/d Adults: <100 mg/d to >500 mg/d	52–156	Seizure response ≥50 reduction: 279 (17.1%) >75% reduction 161 (9.9%) 100% reduction: 636 (39%)
Wilfong et al., 2005, US^25^	Retrospective study	131 patients with focal and generalized seizure/epilepsy types^a^	100 mg/d to maximum 54 mg/kg/d	180	Seizure response 50%–74.9%: 18 (13.7%) 75%–99.9%: 44 (33.6%) 100% reduction: 39 (29.8%)
Absence seizures					
Wilfong et al., 2005, US^26^	Retrospective study	45 patients aged 18 y or younger with absence seizures	Range: 100–600 mg/d or 2–24 mg/kg/d	104	23 (51.1%) achieved freedom from absence seizures
Childhood epilepsies					
Shinnar et al., 2009, US^27^	Phase 3 OLE study	109 patients aged 3–15 y with childhood epilepsies (localized, idiopathic, LGS, infantile epilepsies, other)	1 mg/kg/d to maximum of 12 mg/kg	65	A significant median reduction in “all-seizure” frequency of 2.60 seizures per week was observed (*p* = 0.033)
You et al., 2008, South Korea^28^	Retrospective study	62 patients aged 1–72 mo with LGS	Maximum of 16 mg/kg/d	52	Seizure response >50% to <75% reduction: 15 (24.2%) >75% to <100% reduction: 14 (22.6%) 100% reduction: 3 (4.8%)
Lee et al., 2010, South Korea^29^	Retrospective study	163 patients aged 0.08–13 y with childhood intractable epilepsies (partial, generalized, LGS, infantile epilepsies)	3–5 mg/kg/d titrated to maximum tolerability	104	Seizure response >50% reduction: 79 (48.5%) 100% reduction: 25 (15.3%)
Kothare, 2004, US^30^	Retrospective study	15 patients aged 11–20 y with juvenile myoclonic epilepsy (GTC, myoclonic, or absence seizures)	Range: 200–500 mg/d	8–104	Seizure response (GTC, myoclonic, absence seizures) 50%–74% reduction: 1 (7.5%); 1 (7.5%); 3 (23%) 75%–99% reduction: 1 (7.5%); 1 (7.5%); 2 (15.5%) 100% reduction: 9 (69.5%); 8 (62%); 5 (38.5%)

Abbreviations: BID = twice daily; EU = European Union; GTC = generalized tonic-clonic; ITT = intent to treat; LGS = Lennox-Gastaut syndrome; OLE = open-label extension; RCT = randomized controlled trial; UK = United Kingdom; US = United States; ZNS = zonisamide.

Table is organized by increasing levels of evidence (RCT, OLE following its RCT, prospective study, retrospective study).

a Generalized epilepsies are classified as tonic-clonic, tonic, clonic, atonic, myoclonic, typical absence, and atypical absence seizures.

This article included a total of 16 studies evaluating ZNS efficacy by analyzing responder rates and seizure-free events. Overall, ZNS was administered up to 600 mg/d in adult and pediatric studies. ZNS was associated with significant reductions in seizure frequency and overall improvements in 15 of 16 evaluated studies.

In the existing literature of ZNS, most studies examined patients with focal-onset seizures. Numerous studies of ZNS efficacy are lacking in this review because many are untranslated and published in Japanese.^25^ However, multiple English review articles of Japan-based studies have evaluated postmarketing surveillance studies and phase 2 and phase 3 trials to establish efficacy and safety of ZNS treatment in focal and generalized seizures.^31,32^

ZNS was established as an efficacious treatment in adults with new-onset and refractory focal and generalized epilepsy. In adults with new-onset focal seizures or unclassified GTC seizures, ZNS was shown to have nearly identical seizure-free rates compared with controlled release CBZ (79.4% vs 93.7%, respectively) after 26 weeks (adjusted absolute treatment difference: −4.5%, 95% confidence interval [CI] −12.2 to 3.1).^5^ These findings resulted in a Level C recommendation for ZNS use to decrease seizure frequency in adult and pediatric patients with new-onset, focal-onset, or unclassified GTC seizures.^4^ A randomized, double-blind, placebo-controlled trial conducted in adults aged 18–70 years with refractory focal-onset or secondary generalized seizures revealed that ZNS 300 and 400 mg/d significantly reduced seizure frequency compared with placebo (*p* < 0.05).^17^ No difference in incidence of AEs was observed between the ZNS and placebo groups.

The efficacy of ZNS in pediatric and adult patients with focal and generalized epilepsy was investigated in 8 RCTs, OLEs, chart reviews, and postmarketing studies. A multicenter, double-blind, phase 3 RCT published in 2013 investigated the efficacy, safety, and tolerability of ZNS in pediatric patients (6–17 years) with focal epilepsy.^18^ This study allowed for 2018 AAN/AES guideline Level A recommendations for ZNS as adjunctive treatment of focal epilepsy for this age group.^16^ At a dose of 8 mg/kg/d, ZNS response rates were higher compared with placebo. A slight increase in the rate of some mild side effects (e.g., weight loss and decreased appetite) was observed, but no new or unexpected safety concerns were discovered. An OLE of the 2013 phase 3 RCT study confirmed initial findings that adjunctive ZNS was well-tolerated and efficacious for up to 57 weeks in pediatric patients (6–18 years) with focal epilepsy.^19^ A 2005 RCT and corresponding 2008 OLE study demonstrated efficacy for ZNS 300 and 500 mg/d in patients 12 years or older with refractory focal seizures.^21,22^ Additional studies supported the use of ZNS in reducing seizure frequency for pediatric patients with focal seizures.^23-25^ In the SANAD II study (an open-label RCT of patients 5 years or older), ZNS was deemed noninferior to lamotrigine in time to 12-month remission in patients with new-onset focal seizures.^20^ In all other studies, patients on ZNS exhibited a ≥50% reduction in seizures. ZNS was well-tolerated in these studies, with insomnia, somnolence, dizziness, and nausea occurring most frequently.^5,18-25^

There are a few studies on the efficacy of ZNS in absence seizures and other childhood epilepsies. ZNS blockade of T-type calcium channels has been studied as a key contributor in the treatment of absence seizures. In a chart review of 45 pediatric patients 18 years or younger, 51.1% achieved complete freedom from absence seizures.^26^ Of the 4 studies that assessed other childhood epilepsies, 3 evaluated the use of ZNS in patients up to age 15 years with Lennox-Gastaut syndrome (LGS).^27-29^ ZNS adjunctive therapy was found to be effective and safe in patients with LGS. ZNS has also shown efficacy as a treatment for pediatric patients with atonic or myoclonic seizures, both historically more refractory than other seizure types.

ZNS demonstrates robust and broad spectrum efficacy for generalized and focal seizures and has established efficacy and safety uses for mono/adjunctive therapy in both adults and children. Primary outcome measures of seizure free, responder, and seizure frequency rates all showed significant improvements for ZNS vs placebo.^17-19,21-30^ For RCT and real-world OLE studies of ZNS and comparator drugs (CBZ, LTG), ZNS achieved noninferiority.^5,6,20^ In addition to a proven efficacy record in US Food and Drug Administration (FDA)–approved indications for partial seizures in patients 16 years or older, study results indicate that ZNS has the potential for broad use in non–FDA-approved seizure types, including GTC, absence, and childhood seizures associated with LGS (tonic, atonic, atypical absence, myoclonic, and tonic-clonic).^5,6,17-30^

## Potential Nonepilepsy Uses of ZNS

Preclinical studies with ZNS have demonstrated neuroprotective mechanisms not seen in other ASMs, including free radical scavenging activities, protection against glutamate-induced neuronal damage, and reduction in hypoxic-ischemic brain damage.^3^ These unique effects contribute to the prevention of cerebral infarction and may be useful in movement disorders, such as Parkinson disorder (PD).

In 2009, ZNS was approved in Japan for adjunctive treatment of PD at a dose of 25 or 50 mg/d.^33^ ZNS 25 and 50 mg/d significantly improved motor dysfunction in the Unified Parkinson Disease Rating Scale part III (UPDRS-III) up to 28 weeks (mean [SD] change from baseline was −5.1 [7.3] and −6.3 [8.2], respectively; *p* < 0.001). ZNS 25 mg was even found to exert clinically relevant improvements in the UPDRS-III score as compared with a 50 mg dose, which may benefit patients who might need a dose reduction for any reason.

Effects of ZNS on the dopaminergic system and inhibition of T-type calcium channels may also lead to clinical improvements in sleep disorders, pain, and migraine.^34^ Studies in animal and disease state models revealed that ZNS enables motor function recovery and decreased muscle atrophy through multiple pathways. In patients with migraines and intolerance to topiramate, ZNS may be an effective alternative therapy.^35^

ZNS may positively contribute to the management of comorbid disorders such as diabetes and obesity as well as overall cardiovascular disorders. Animal model studies revealed that ZNS treatment of 40 mg/kg/d for 16 weeks has a protective effect against type 2 diabetes mellitus (T2DM) complications.^36,37^ Finally, ZNS is associated with weight loss; however, patients with comorbid obesity may use this side effect to their advantage.^38,39^ Patients on ZNS up to 400 mg/d significantly reduced their body weight compared with placebo, without a significant impact on bone mineral density. ZNS may also improve risk factors associated with obesity.

In addition, ZNS modulates the neuronal release of dopamine and serotonin, which may lead to a reduction in alcohol consumption.^40^ In a double-blind, randomized controlled trial of patients with alcohol dependency, ZNS 500 mg/d resulted in a decrease in the urge to drink compared with the placebo group. Alcohol used disorder (AUD) is also commonly experienced in individuals with post-traumatic stress disorder (PTSD).^41^ A 12-week study of veterans diagnosed with AUD and PTSD revealed that drinking significantly decreased in patients taking ZNS 400 mg/d. ZNS shows promising results individuals struggling with alcohol overuse, although further studies are warranted.

## Safety Considerations/Adverse Effects

In a 2018 Japanese retrospective cohort study, VPA was the most widely prescribed older (approved before 1990) ASM (43.3%), whereas ZNS made up only 9.2% of total older ASM prescriptions.^42^ Newer ASMs saw an upward trend in prescribing rates from 2015 to 2018, possibly due to perceived fewer AEs and drug interactions compared with older ASMs, although newer ASMs may not offer better overall seizure control. A review of common concerns from providers on the AEs and conceived limitations of ZNS follows.

### Adverse Effects

ZNS is generally well-tolerated, with common adverse reactions (>4% vs placebo) of somnolence, anorexia, dizziness, ataxia, agitation/irritability, and difficulty with memory and/or concentration.^43^ These tend to be dose and time dependent. The potentially more frequent and serious AEs that occur more frequently than placebo are discussed below.

The sulfamoyl group in the ZNS molecule may inhibit carbonic anhydrase activity in the brain.^1,3^ The systemic effect of carbonic anhydrase inhibitor activity is associated with the production of metabolic acidosis.^2,14^ This effect is usually mild; however, regular testing of serum bicarbonate should be conducted in patients with impaired pulmonary or renal function. This effect could be exacerbated by coadministration with topiramate or the ketogenic diet. In addition, fatalities have occurred because of reactions to sulfonamides, including Stevens-Johnson syndrome, toxic epidermal necrolysis, fulminant hepatic necrosis, agranulocytosis, aplastic anemia, and other blood dyscrasias, so ZNS should be discontinued at first sign of a rash.^43^ In US and European RCTs, 2.2% of ZNS patients discontinued treatment due to rash. Other warnings associated with ZNS include serious hematologic events, drug reaction with eosinophilia and systemic symptoms/multiorgan sensitivity, oligohydrosis and hyperthermia in pediatric patients, acute myopia and secondary angle-closure glaucoma, suicidal behavior and ideation, and teratogenicity. The chemical structure of ZNS contains a sulfonamide side chain, and as such, it has been suggested that there may be hypersensitivity cross-reactivity with other non-ASM sulfonamides, such as sulfa-based antibiotics. However, there is a lack of clinical data to support this observation.

ASMs may have teratogenic potential.^2^ PHT, VPA, and TPM are all associated with anatomical or behavioral teratogenicity. VPA has been associated with dose-dependent adaptive behavior impairments, leading to a US FDA pregnancy categorization of X (contraindicated).^44^ Topiramate was also shown to increase major congenital malformation risks and is grouped as a category D medication (evidence of risk). Some studies suggest that the teratogenic risk associated with ZNS may be low. A Dutch study examining the use of ZNS during pregnancy gathered data from 6 different hospitals and concluded that mean gestational times, head circumferences, and birth weights of children born were all within appropriate reference ranges.^45^ A 2012 study using data from the North American ASM Pregnancy Registry found that the risk of congenital malformations when using ZNS during pregnancy was inconclusive compared with older ASMs such as VPA and phenobarbital.^46^ Overall, ZNS has a pregnancy categorization of C (caution). More data from diverse populations are needed to confirm ZNS's safety in pregnancy.

Existing literature provides evidence suggesting that ZNS treatment may be associated with decreased sweating, appetite, and weight, particularly at higher doses. More than 25% of children taking ZNS for drug-resistant epilepsy reported (n/N = 28/109) decreased appetite in an open-label study conducted during 2009.^27^ Decreased appetite was reported most frequently in the youngest age group (3–4 years). A large RCT conducted in 2013 found that both weight and appetite loss in children with focal epilepsy were more common for those taking ZNS compared with placebo.^18^ The reports of decreased weight and decreased appetite for patients prescribed ZNS were higher compared with placebo. These studies revealed associations between ZNS treatment and decreased appetite/weight loss.

Other studies demonstrated a potential correlation between ZNS treatment and cognitive dysfunction. A 2007 prospective, randomized, open-label investigation reported that nearly half of the patients complained of cognitive problems after 1 year of ZNS treatment (n/N = 8/16).^47^ Memory deficit (35%), attention/concentration deficit (26%), speech problems (12%), and dyscalculia (6%) were the most common cognitive-related complaints. Daily ZNS administration significantly affected cognitive scores, including verbal fluency, Trail Making Test Part B, and delayed word recall. Multiple studies have provided evidence to support that long-term cognitive effects of ZNS (e.g., worsened memory, concentration, mood, anxiety, and language ability) are more likely associated with higher doses.^47,48^

Decreased sweating (oligohydrosis) is another common negative effect observed with ZNS treatment.^49^ The mechanism of oligohydrosis is not fully understood, but it is believed to be caused by the carbonic anhydrase inhibitor properties of ZNS. Cases of oligohydrosis have been reported in Japan for decades, but one study conducted in the United States identified 6 reported cases of oligohydrosis and/or fever associated with ZNS between March 2000 and 2001. The occurrence of ZNS-associated oligotrophy was shown to be infrequent and is commonly noted in pediatric patients. Current literature also suggests that age and dosing may significantly affect the prevalence of these negative effects.

Renal calculi development is rare but more common in patients taking ZNS for at least 6 months.^50^ In US and European studies, the onset of symptomatic nephrolithiasis was observed in patients with history of renal calculi.^1^ In total, 3.4% of patients in these studies developed renal calculi throughout the study period. It is recommended that patients maintain adequate hydration to decrease the risk of renal calculi and to maintain appropriate urine flow.^50^

Overall, the AE profiles of available older and newer-generation ASMs are similar.^4,11,16,51^ Neurologic, laboratory, metabolic, pregnancy, congenital, and weight-based changes are seen in the usage of many ASMs. ZNS has an established safety profile with common and documented side effects and known mitigation strategies. Its use is relatively safe and does not have AEs evident with other ASMs, including tremor, weight gain, thrombocytopenia, pancreatitis, and reproductive disorders (associated with VPA), or an increased rate of aggression (associated with LEV and PER).^2,4,16,52^ With its clinical use commencing in 1989, new or severe idiosyncratic reactions due to ZNS use would not be expected. The AEs for approved ASM treatments and their MOAs are summarized in Table 4.^11,43,51^

**Table 4:**
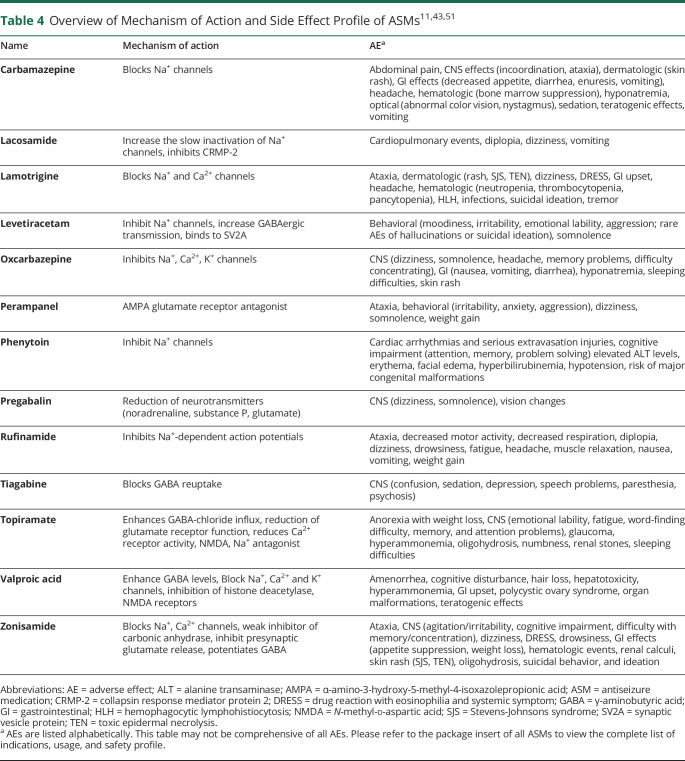
Overview of Mechanism of Action and Side Effect Profile of ASMs^11,43,51^

Name	Mechanism of action	AE^a^
Carbamazepine	Blocks Na^+^ channels	Abdominal pain, CNS effects (incoordination, ataxia), dermatologic (skin rash), GI effects (decreased appetite, diarrhea, enuresis, vomiting), headache, hematologic (bone marrow suppression), hyponatremia, optical (abnormal color vision, nystagmus), sedation, teratogenic effects, vomiting
Lacosamide	Increase the slow inactivation of Na^+^ channels, inhibits CRMP-2	Cardiopulmonary events, diplopia, dizziness, vomiting
Lamotrigine	Blocks Na^+^ and Ca^2+^ channels	Ataxia, dermatologic (rash, SJS, TEN), dizziness, DRESS, GI upset, headache, hematologic (neutropenia, thrombocytopenia, pancytopenia), HLH, infections, suicidal ideation, tremor
Levetiracetam	Inhibit Na^+^ channels, increase GABAergic transmission, binds to SV2A	Behavioral (moodiness, irritability, emotional lability, aggression; rare AEs of hallucinations or suicidal ideation), somnolence
Oxcarbazepine	Inhibits Na^+^, Ca^2+^, K^+^ channels	CNS (dizziness, somnolence, headache, memory problems, difficulty concentrating), GI (nausea, vomiting, diarrhea), hyponatremia, sleeping difficulties, skin rash
Perampanel	AMPA glutamate receptor antagonist	Ataxia, behavioral (irritability, anxiety, aggression), dizziness, somnolence, weight gain
Phenytoin	Inhibit Na^+^ channels	Cardiac arrhythmias and serious extravasation injuries, cognitive impairment (attention, memory, problem solving) elevated ALT levels, erythema, facial edema, hyperbilirubinemia, hypotension, risk of major congenital malformations
Pregabalin	Reduction of neurotransmitters (noradrenaline, substance P, glutamate)	CNS (dizziness, somnolence), vision changes
Rufinamide	Inhibits Na^+^-dependent action potentials	Ataxia, decreased motor activity, decreased respiration, diplopia, dizziness, drowsiness, fatigue, headache, muscle relaxation, nausea, vomiting, weight gain
Tiagabine	Blocks GABA reuptake	CNS (confusion, sedation, depression, speech problems, paresthesia, psychosis)
Topiramate	Enhances GABA-chloride influx, reduction of glutamate receptor function, reduces Ca^2+^ receptor activity, NMDA, Na^+^ antagonist	Anorexia with weight loss, CNS (emotional lability, fatigue, word-finding difficulty, memory, and attention problems), glaucoma, hyperammonemia, oligohydrosis, numbness, renal stones, sleeping difficulties
Valproic acid	Enhance GABA levels, Block Na^+^, Ca^2+^ and K^+^ channels, inhibition of histone deacetylase, NMDA receptors	Amenorrhea, cognitive disturbance, hair loss, hepatotoxicity, hyperammonemia, GI upset, polycystic ovary syndrome, organ malformations, teratogenic effects
Zonisamide	Blocks Na^+^, Ca^2+^ channels, weak inhibitor of carbonic anhydrase, inhibit presynaptic glutamate release, potentiates GABA	Ataxia, CNS (agitation/irritability, cognitive impairment, difficulty with memory/concentration), dizziness, DRESS, drowsiness, GI effects (appetite suppression, weight loss), hematologic events, renal calculi, skin rash (SJS, TEN), oligohydrosis, suicidal behavior, and ideation

Abbreviations: AE = adverse effect; ALT = alanine transaminase; AMPA = α-amino-3-hydroxy-5-methyl-4-isoxazolepropionic acid; ASM = antiseizure medication; CRMP-2 = collapsin response mediator protein 2; DRESS = drug reaction with eosinophilia and systemic symptom; GABA = γ-aminobutyric acid; GI = gastrointestinal; HLH = hemophagocytic lymphohistiocytosis; NMDA = *N*-methyl-d-aspartic acid; SJS = Stevens-Johnsons syndrome; SV2A = synaptic vesicle protein; TEN = toxic epidermal necrolysis.

a AEs are listed alphabetically. This table may not be comprehensive of all AEs. Please refer to the package insert of all ASMs to view the complete list of indications, usage, and safety profile.

### Prescribing Zonisamide Dosing Considerations

Zonegran (zonisamide) is FDA approved as adjunctive therapy for the treatment of focal seizures in patients aged 16 years or older with epilepsy.^43^ ZNS 25 mg or 100 mg capsules should be administered once/twice daily and may be titrated up to 400 mg. Zonisade (zonisamide oral suspension 100 mg/5 mL) is approved as adjunctive therapy for the treatment of focal-onset seizures in adults and pediatric patients 16 years or older.^53^ Initial doses of Zonisade 100 mg daily are recommended, which may be titrated by 100 mg every 2 weeks to a daily dose of 400 mg. Patients who tolerate Zonisade 400 mg daily may have up to a maximum of 600 mg daily if they require further reduction of seizures.

In clinic settings, children <50 kg with any seizure types are typically initiated on ZNS 1–2 mg/kg/d in 1–2 divided doses. Their dose may be increased by 1–2 mg/kg/d every 1–2 weeks for a dose range of 4–8 mg/kg/d (maximum 12 mg/kg/d). Typically, preschool-aged children (younger than 5 years) or patients on enzyme inducing medications will require higher doses. It is recommended to use serum levels and clinical responses to guide dosing in all ages.

Because ZNS is metabolized in the liver and excreted in the kidneys, patients with renal or hepatic diseases may require slower titration and more frequent monitoring.^43^ Several variables may predispose patients to higher risks of AEs. Drug-drug interactions, pregnancy, age, and dose strength are factors associated with changes in PK profiles and an increased risk of AEs. The use of ZNS is contraindicated in patients with hypersensitivity to sulfonamides.

Increased dosing is correlated with an increased risk for AEs associated with ZNS. A prospective, randomized, open-label investigation in 2007 concluded that the dose of ZNS significantly affected cognitive function.^47^ Evidence suggests that younger patients or patients on higher doses of ZNS may also be at higher risk of AEs secondary to ZNS.^48^ Further studies and analyses are needed to fully understand the relationship between ZNS and various risk factors associated with increased AEs. Proper awareness of these potential risk factors and careful patient management strategies (such as slow dose titration) are essential for mitigating any negative impacts of these risk factors and ensuring patient safety.^54^

### ASM Formulations

Formulation is an important consideration for prescribers when choosing an ASM, particularly in children and/or older patients with swallowing difficulties.^55^ Prescribers should consider factors such as seizure recurrence, patient age, epilepsy syndromes, drug reactions, and prognosis of the epilepsy syndrome when prescribing an ASM. Patients receiving medications through gastronomy or gastrojejunostomy tubes may benefit from liquid formulations because crushed solid dosage forms can clog tubes. The pediatric population may also prefer nonsolid oral dosage forms for easier ingestion.^56^ Liquid formulations allow more precise dosing to maximize efficacy and decrease AEs. Moreover, age-related health complications in the older patients, such as dysphagia, may require alternate or modified dosage forms.^57^ For patients who have difficulty swallowing, Zonisade was FDA approved in 2022 as an oral liquid formulation alternative to ZNS. ZNS is also available in a powder and orally dispersible tablet formulations. Oral solid dosage forms of ZNS (such as tablets or capsules) may be compounded into alternative liquid formulations. A summary of ZNS formulations and their indications across the globe is presented in Table 5.^3,43,53,58-61^

**Table 5:**
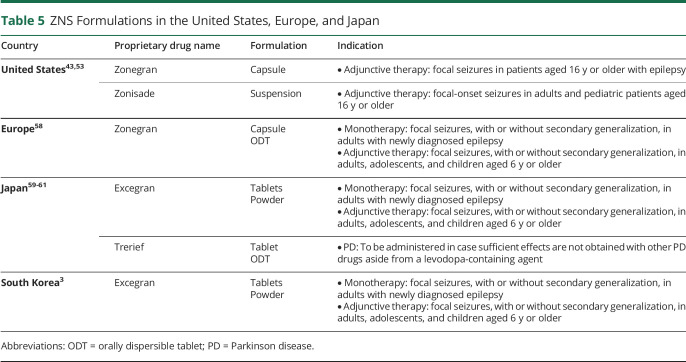
ZNS Formulations in the United States, Europe, and Japan

Country	Proprietary drug name	Formulation	Indication
United States^43,53^	Zonegran	Capsule	• Adjunctive therapy: focal seizures in patients aged 16 y or older with epilepsy
	Zonisade	Suspension	• Adjunctive therapy: focal-onset seizures in adults and pediatric patients aged 16 y or older
Europe^58^	Zonegran	Capsule ODT	• Monotherapy: focal seizures, with or without secondary generalization, in adults with newly diagnosed epilepsy • Adjunctive therapy: focal seizures, with or without secondary generalization, in adults, adolescents, and children aged 6 y or older
Japan^59-61^	Excegran	Tablets Powder	• Monotherapy: focal seizures, with or without secondary generalization, in adults with newly diagnosed epilepsy • Adjunctive therapy: focal seizures, with or without secondary generalization, in adults, adolescents, and children aged 6 y or older
	Trerief	Tablet ODT	• PD: To be administered in case sufficient effects are not obtained with other PD drugs aside from a levodopa-containing agent
South Korea^3^	Excegran	Tablets Powder	• Monotherapy: focal seizures, with or without secondary generalization, in adults with newly diagnosed epilepsy • Adjunctive therapy: focal seizures, with or without secondary generalization, in adults, adolescents, and children aged 6 y or older

Abbreviations: ODT = orally dispersible tablet; PD = Parkinson disease.

## Expert Suggestions for Practical Use/Clinical Pearls

ZNS was associated with significant reductions in seizure frequency and overall improvements in most evaluated studies. ZNS was administered in most studies as a once-daily dosing schedule.^4^ Its long t_1/2_ of up to 3 days also supports once-daily dosing, potentially leading to improved adherence compared with other ASMs with more frequent administration.^10^ ZNS is generally well-tolerated with known and manageable safety issues and multiple MOAs contributing to antiepileptic activity, i.e., targeting Na^+^, T-type Ca^2+^ channels, and modulating glutamate and GABA function.^8^ This allows for coadministration with ASMs of different MOAs to increase medication efficacy and reduce seizure frequency. The linear dose-response relationship of ZNS drug concentration to serum levels also means that efficacy can improve significantly with dose.^1^ Studies have demonstrated that ZNS has no clinically significant effects on the PK profile of other common ASMs, indicating that coadministration of ZNS and other ASMs is safe.^54^ However, because ZNS is partially metabolized by cytochrome P450 3A4 (CYP3A4), both CYP3A4 inducers and inhibitors may affect ZNS PK. Coadministration of ZNS with CYP3A4 inducers was associated with lower ZNS concentrations, which may lead to lower efficacy. Conversely, higher plasma concentrations can increase the risk of AEs.

ZNS is an older, broad spectrum ASM with unique MOAs contributing to potential efficacy in therapeutic areas outside epilepsy, compared with other ASMs. ZNS is used and approved in multiple countries for the treatment of focal-onset seizures for adults and children but has also demonstrated efficacy in LGS and absence seizures. Although there are potential concerns for bone-related AEs, negative effects (e.g., sweating, kidney stones, cognitive dysfunction), and teratogenicity, literature findings revealed that many of these effects may be dose dependent, or studies have shown inconsistent results. These AEs may also be associated with older-generation and newer-generation ASMs. ZNS is a safe and effective ASM that has therapeutic indications in multiple epilepsy etiologies and may have potential benefits in other disease states. ZNS may not be frequently thought of as the first-line ASM in the treatment landscape of epilepsy, possibly due to a mischaracterization of safety or because of a lack of marketing. However, there are many benefits to using ZNS in the clinical setting. Multiple available formulations, a once-daily dosing regimen, and a recognized safety profile allow use of this drug in patient populations in which therapeutic regimens need to be optimized for adherence, ease of use, and quality of life.

## Appendix Authors

**Table:**

Name	Location	Contribution
Barry E. Gidal, PharmD	Pharmacy Practice & Translational Research, University of Wisconsin-Madison	Drafting/revision of the manuscript for content, including medical writing for content; major role in the acquisition of data; study concept or design; analysis or interpretation of data
Trevor Resnick, MD	Department of Neurology, Nicklaus Children Hospital; Department of Neurology, Florida International University.	Drafting/revision of the manuscript for content, including medical writing for content; major role in the acquisition of data; study concept or design; analysis or interpretation of data.
Michael C. Smith, MD	Department of Neurological Sciences, RUSH Medical College; Rush University Medical Center	Drafting/revision of the manuscript for content, including medical writing for content; major role in the acquisition of data; study concept or design; analysis or interpretation of data
James W. Wheless, BScPharm, MD	Pediatric Neurology, University of Tennessee Health Science Center; Neuroscience Institute & Le Bonheur Comprehensive Epilepsy Program, Le Bonheur Children's Hospital	Drafting/revision of the manuscript for content, including medical writing for content; major role in the acquisition of data; study concept or design; analysis or interpretation of data
